# Enantioselective Michael addition of 2-hydroxy-1,4-naphthoquinones to nitroalkenes catalyzed by binaphthyl-derived organocatalysts

**DOI:** 10.3762/bjoc.8.78

**Published:** 2012-05-07

**Authors:** Saet Byeol Woo, Dae Young Kim

**Affiliations:** 1 Department of Chemistry, Soonchunhyang University, Asan, Chungnam, 336-745, Korea

**Keywords:** asymmetric catalysis, Michael addition, 1,4-naphthoquinones, nitroalkenes, organocatalysis

## Abstract

The highly enantioselective Michael addition of 2-hydroxy-1,4-naphthoquinones to nitroalkenes, promoted by binaphthyl-modified chiral bifunctional organocatalysts is described. This reaction afforded the chiral functionalized naphthoquinones in high yields (81–95%) and excellent enantioselectivities (91–98% ee) under low catalyst loading (1 mol %).

## Introduction

Quinone and naphthoquinone structures exist in a large number of natural products and biologically active molecules [1–4]. Many of these naturally occurring naphthoquinones and their synthetic analogues are important precursors for the synthesis of natural products and pharmaceuticals [5–9]. The stereoselective formation of C–C bonds is of great importance for the synthesis of enantiomerically pure, biologically active organic compounds [10–11]. It is widely recognized that the Michael addition is one of the most versatile and general methods for C–C bond formation in organic synthesis [12], and intensive research efforts have been directed toward the development of enantioselective catalytic protocols for this reaction [13–15]. The organocatalyst-mediated enantioselective conjugate addition reactions, which are both powerful and environmentally friendly, have been subjected to rigorous investigation in recent years [16–22]. The asymmetric Michael addition of various nucleophiles to nitroalkenes is of great interest, because the products obtained are versatile intermediates in organic synthesis [23–26]. Extensive studies have been devoted to the development of asymmetric conjugate additions of 1,3-dicarbonyl compounds to various Michael acceptors [27–33]. Recently, the groups of Du and Zhou reported a highly enantioselective Michael addition of 2-hydroxy-1,4-naphthoquinones to nitroalkenes catalyzed by chiral, bifunctional tertiary-amine thioureas, thiophosphorodiamides, and squaramide-based organocatalysts [34–36].

## Findings

**Figure 1:**
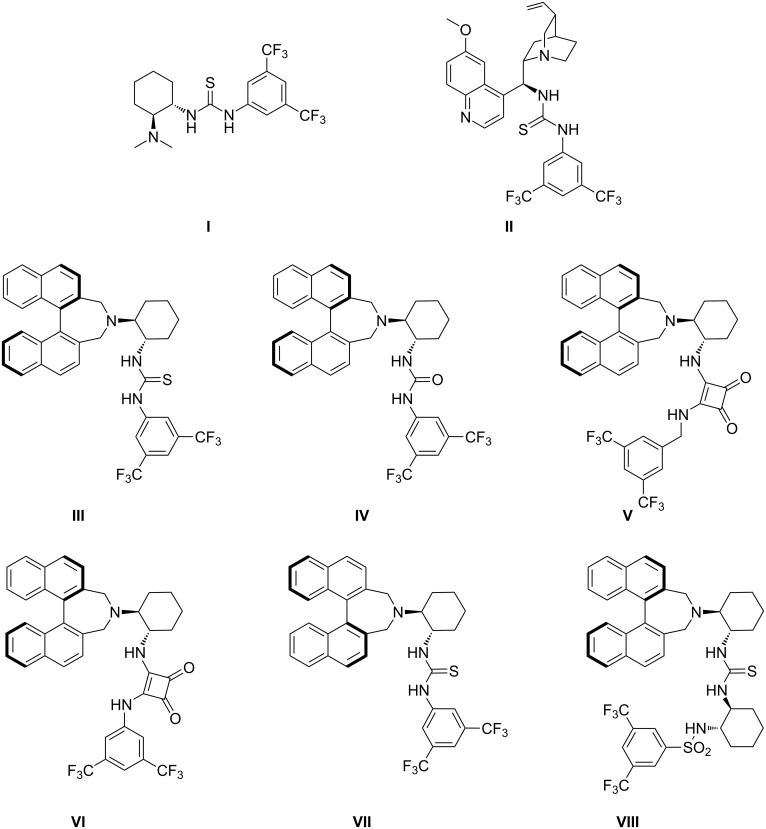
Structures of chiral organocatalysts.

In the framework of our research program for the development of synthetic methods for the enantioselective construction of stereogenic carbon centers [37–42], we recently reported the enantioselective Michael addition of active methines to nitroalkenes [43–44]. Herein, we describe the direct enantioselective Michael addition of 2-hydroxy-1,4-naphthoquinone with nitroalkenes, catalyzed by bifunctional organocatalysts (Figure 1) that bear both central and axial chiral elements [45–47].

We initially investigated the reaction system with 2-hydroxy-1,4-naphthoquinone (**1**) and nitrostyrene **2a** in the presence of 10 mol % of Takemoto's catalyst **I** in acetonitrile at room temperature, to determine the optimum reaction conditions for the catalytic, enantioselective Michael addition. This reaction exhibited good yield and high enantioselectivity (89% ee, Table 1, entry 1). In order to enhance the enantioselectivity, other bifunctional organocatalysts **II**–**VIII** were evaluated in the model reaction (Table 1, entries 2–8). The quinine-derived thiourea catalyst **II** was less effective (Table 1, entries 1 and 2), whereas the binaphthyl-modified, chiral, bifunctional organocatalysts **III**–**VIII**, bearing both central and axial chiral elements, effectively promoted the addition reaction in high yield, with high enantioselectivity (78–97% ee, Table 1, entries 3–8). Catalyst **III** gave the desired product **3a** with high enantioselectivity (97%, Table 1, entry 3), whereas the diastereomeric catalyst **VII** afforded product **3a** in lower enantioselectivity (78% ee, Table 1, entry 7). These results demonstrate that the central and axial chiral elements in the chiral amine-thiourea catalyst **III** are matched, thus enhancing the stereochemical control, whereas in the diastereomeric catalyst **VII** this is not the case.

**Table 1:** Optimization of the reaction conditions.

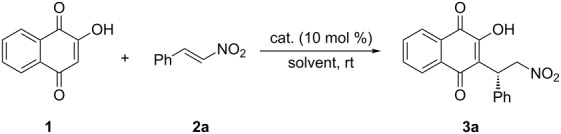					

entry	cat.	solvent	time (h)	yield (%)^a^	ee (%)^b^

1	**I**	CH_3_CN	2	84	89
2	**II**	CH_3_CN	2	87	77
3	**III**	CH_3_CN	2	96	97
4	**IV**	CH_3_CN	2	95	87
5	**V**	CH_3_CN	2	93	81
6	**VI**	CH_3_CN	2	90	93
7	**VII**	CH_3_CN	2	85	78
8	**VIII**	CH_3_CN	2	88	93
9	**III**	toluene	4	75	95
10	**III**	DCM	4	93	89
11	**III**	THF	2	92	99
12	**III**	Et_2_O	3	81	91
13	**III**	H_2_O	17	89	19
14	**III**	brine	17	86	37
15^c^	**III**	THF	2	90	98
16^d^	**III**	THF	2	90	99
17^e^	**III**	THF	2	89	99

^a^Isolated yield. ^b^Enantiopurity was determined by HPLC analysis using chiralcel OJ-H column. ^c^Reaction was carried out in the presence of 5 mol % catalyst. ^d^Reaction was carried out in the presence of 2.5 mol % catalyst. ^e^Reaction was carried out in the presence of 1 mol % catalyst.

Different solvents were then tested in the presence of 10 mol % of catalyst **III** together with 2-hydroxy-1,4-naphthoquinone (**1**) and nitrostyrene **2a** in order to further improve the selectivity of the reaction. Aprotic solvents, such as acetonitrile, toluene, dichloromethane, THF, diethyl ether, were well tolerated in this conjugate addition without a significant decrease of enantioselectivities (89–99% ee, Table 1, entries 3 and 9–12). Remarkably, water and brine also afforded products in good yields; however, the selectivity dropped significantly (Table 1, entries 13 and 14). Among the solvents probed, the best results (92% yield and 99% ee) were achieved when the reaction was conducted in THF (Table 1, entry 11). The present catalytic system tolerates catalyst loading down to 5, 2.5, and 1 mol % without compromising the yield or enantioselectivity (Table 1, entries 11 and 15–17).

With the optimized reaction conditions in hand, the scope of the methodology was investigated in reactions with 2-hydroxy-1,4-naphthoquinone (**1**) and various nitroalkenes **2a**–**l** in the presence of 1 mol % of catalyst **III** in THF at room temperature (Table 2). A range of electron-donating and electron-withdrawing substitutions on the β-aryl ring of the nitrostyrenes **2b**–**h** provided reaction products in high yields and excellent enantioselectivities. Heteroaryl- and naphthyl-substituted nitroalkenes **2i** and **2j** provided products with high selectivity (93–99% ee, Table 2, entries 9 and 10). The β-alkyl-substituted nitroalkene, 4-methyl-1-nitropent-1-ene (**2k**), was also an acceptable starting material and provided the corresponding Michael adducts in high yield and excellent enantioeselectivity (97% ee, Table 2, entry 11).

**Table 2:** Catalytic asymmetric Michael addition of 2-hydroxy-1,4-naphthoquinone **1** to nitroalkenes **2**.

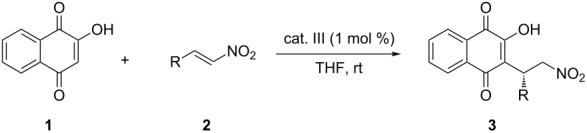				

entry	**2**, R	time (h)	yield (%)^a^	ee (%)^b^

1	**2a**, Ph	2	**3a**, 89	99
2	**2b**, *p*-MeC_6_H_4_	2	**3b**, 93	95
3	**2c**, *p*-MeOC_6_H_4_	4	**3c**, 81	99
4	**2d**, *p*-FC_6_H_4_	3	**3d**, 95	95
5	**2e**, *p*-ClC_6_H_4_	3	**3e**, 90	91
6	**2f**, *p*-BrC_6_H_4_	3	**3f**, 95	95
7	**2g**, *o*-FC_6_H_4_	4	**3g**, 95	95
8	**2h**, *o*-BrC_6_H_4_	4	**3h**, 95	95
9	**2i**, 2-thienyl	5	**3i**, 93	93
10	**2j**, 2-naphthyl	5	**3j**, 93	99
11	**2k**, isobutyl	5	**3k**, 90	97

^a^Isolated yield. ^b^Enantiopurity was determined by HPLC analysis using chiralcel OJ-H (**3a**–**j**) and chiralpak AD-H (for **3k**) columns.

In conclusion, we have developed a highly efficient catalytic, enantioselective Michael addition of 2-hydroxy-1,4-naphthoquinone to nitroalkenes using a binaphthyl-derived tertiary amine-thiourea organocatalyst. The various types of nitroalkylated naphthoquinone derivatives were obtained in good to high yields with excellent enantioselectivities (91–99% ee) for all the substrates examined in this work. We believe that this method should provide a practical entry for the preparation of chiral nitroalkylated naphthoquinone derivatives. Further details and application of this asymmetric Michael addition of 2-hydroxy-1,4-naphthoquinone nucleophiles will be presented in due course.

## Experimental

**General procedure for the Michael addition of 2-hydroxy-1,4-naphthoquinone (1) with nitroalkenes 2:** A mixture of 2-hydroxy-1,4-naphthoquinones (**1**, 34.8 mg, 0.2 mmol) and catalyst **III** (1.3 mg, 0.002 mmol) in THF (0.4 mL) was stirred at room temperature for 5 min. A solution of nitroalkene **2** (0.2 mmol) was added. The reaction mixture was stirred for 2–5 h at room temperature. After completion of the reaction, the resulting solution was concentrated in vacuo and the obtained residue was purified by flash chromatography (EtOAc–hexane) to afford the corresponding Michael adducts **3**. Products **3** are known compounds, and their data were identical to those reported in the literature [34–36].

## Supporting Information

File 1 Characterization data of products **3**.
