# Supplementary material for: Enantioselective Michael addition of 2-hydroxy-1,4-naphthoquinones to nitroalkenes catalyzed by binaphthyl-derived organocatalysts
Source: Beilstein J Org Chem. 2012 May 7;8:699–704. doi: 10.3762/bjoc.8.78 (PMC3388856; doi:10.3762/bjoc.8.78)
Supplement: File 1 — Characterization data of products 3. [file Beilstein_J_Org_Chem-08-699-s001.pdf]

Supporting Information

for

Enantioselective Michael addition of 2-hydroxy-1,4-naphthoquinones to nitroalkenes catalyzed by binaphthyl-derived organocatalyst

Saet Byeol Woo and Dae Young Kim*

Address: Department of Chemistry, Soonchunhyang University, Asan, Chungnam, 336-745, Korea

Email: Dae Young Kim* - dyoung@sch.ac.kr

* Corresponding author

Characterization data of products 3

(*R*)-2-Hydroxy-3-(2-nitro-1-phenylethyl)-1,4-naphthoquinone (3a)

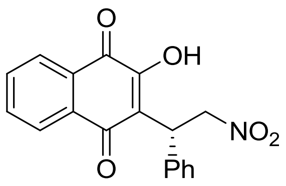

$[\alpha]^{17}_{D}$ = –44.8 (c = 1.00, CH_3COCH_3); mp 153–155 °C; ^1H NMR (200 MHz, $CDCl_3$) δ 8.19–7.99 (m, 2H), 7.83–7.62 (m, 3H), 7.58-7.41 (m, 2H), 7.39–7.21 (m, 2H), 5.47 (dd, *J* = 8.7 Hz, 13.0 Hz, 1H), 5.35–5.27 (m, 1H), 5.14 (dd, *J* = 7.8 Hz, 13.0 Hz, 1H); ^{13}C NMR (50 MHz, $CDCl_3$): δ 39.7, 76.4, 120.7, 126.4, 127.2, 127.9, 128.4, 129.0, 132.7, 133.4, 135.5, 137.6, 153.3, 181.1, 183.8; HPLC (*n*-Hexane/iPrOH = 70/30, 254 nm, 1.0 mL/min) Chiralcel OJ-H, t_R = 27.1 min (minor), t_R = 44.6 min (major), 99% ee.

(*R*)-2-Hydroxy-3-[1-(4-methylphenyl)-2-nitroethyl]-1,4-naphthoquinone (3b)

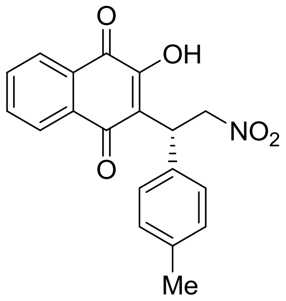

$[\alpha]^{17}_{D}$ = –33.8 (c = 1.00, CH_3COCH_3); mp 163–166 °C; ^1H NMR (200 MHz, $CDCl_3$) δ 8.17–8.01 (m, 2H), 7.83–7.62 (m, 2H), 7.35 (d, *J* = 7.8 Hz, 2H), 7.12 (d, *J* = 7.8 Hz, 2H), 5.47 (dd, *J* = 7.8 Hz, 13.0 Hz, 1H), 5.31–5.24 (m, 1H), 5.12 (dd, *J* = 7.8 Hz, 13.0 Hz, 1H), 2.29 (s, 3H); ^{13}C NMR (50 MHz, $CDCl_3$): δ 21.0, 39.4, 76.5, 120.9, 126.4, 127.3, 128.2, 128.9, 129.6, 132.7, 133.3, 134.5, 135.4, 137.7, 153.1, 181.2, 183.8; HPLC (*n*-Hexane/iPrOH = 70/30, 254 nm, 1.5 mL/min) Chiralcel OJ-H, t_R = 13.2 min (minor), t_R = 40.2 min (major), 95% ee.

(*R*)-2-Hydroxy-3-[1-(4-methoxyphenyl)-2-nitroethyl]-1,4-naphthoquinone (3c)

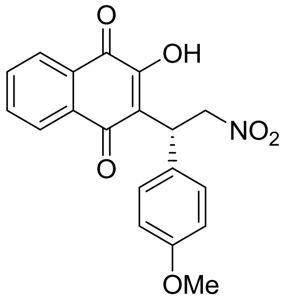

$[\alpha]^{17}_{D}$ = –23.4 (c = 1.00, CH_3COCH_3); mp 165–167 °C; ^1H NMR (200 MHz, $CDCl_3$) δ 8.09–8.00 (m, 2H), 7.80 iPrOH 7.69 (m, 3H), 7.42 (d, *J* = 8.7 Hz, 2H), 6.79 (d, *J* = 8.7 Hz, 2H), 5.40–5.33 (m, 1H), 5.26–5.21 (m, 2H), 3.73 (s, 3H); ^{13}C NMR (50 MHz, $CDCl_3$): δ 38.9, 55.3, 76.6, 114.4, 121.1, 126.4, 127.2, 129.0, 129.4, 132.5, 133.3, 135.4, 153.0, 159.2, 181.2,

183.8; HPLC (*n*-Hexane/iPrOH = 70:30, 254 nm, 1.5 mL/min) Chiralcel OJ-H, t_R = 28.4 min (minor), t_R = 55.1 min (major), 99% ee.

(*R*)-2-[1-(4-Fluorophenyl)-2-nitroethyl]-3-hydroxy-1,4-naphthoquinone (3d)

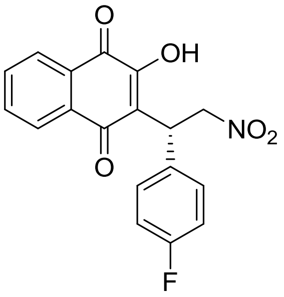

$[\alpha]^{17}_D$ = –36.9 (c = 1.00, CH_3COCH_3); mp 158–159 °C; ^{1}H NMR (200 MHz, $CDCl_3$) δ 8.15–8.03 (m, 2H), 7.92–7.63 (m, 2H), 7.50–7.37 (m, 2H), 7.04–6.95 (m, 2H), 5.41 (dd, *J* = 8.9 Hz, 13.0 Hz, 1H), 5.32–5.25 (m, 1H), 5.14 (dd, *J* = 8.0 Hz, 13.0 Hz, 1H); ^{13}C NMR (50 MHz, $CDCl_3$): δ 38.9, 76.3, 115.9, 116.1, 120.5, 126.6, 126.9, 128.9, 129.9, 130.0, 132.5, 133.3, 133.4, 135.5, 153.1, 162.2 (d, *J* = 244.3 Hz), 181.1, 183.6; HPLC (*n*-Hexane/iPrOH = 70/30, 254 nm, 1.5 mL/min) Chiralcel OJ-H, t_R = 8.0 min (minor), t_R = 27.5 min (major), 97% ee.

(*R*)-2-[1-(4-Chlorophenyl)-2-nitroethyl]-3-hydroxy-1,4-naphthoquinone (3e)

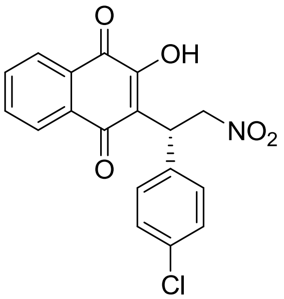

$[\alpha]^{17}_D$ = –27.3 (c = 1.00, CH_3COCH_3); mp 193–197 °C; ^{1}H NMR (200 MHz, $CDCl_3$) δ 8.05 (d, *J* = 7.8 Hz, 1H), 7.92 (d, *J* = 6.8 Hz, 1H), 7.72 (t, *J* = 7.8 Hz, 1H), 7.61–7.50 (m, 3H), 7.07 (d, J = 8.7 Hz, 2H), 5.57–5.46 (m, 1H), 5.33–5.18 (m, 2H); ^{13}C NMR (50 MHz, $CDCl_3$): δ 37.7, 76.3, 120.0, 125.7, 125.9, 128.4, 129.7, 129.8, 131.6, 131.7, 133.4, 134.6, 137.5, 156.8, 180.8, 183.5; HPLC (*n*-Hexane/iPrOH = 70/30, 254 nm, 1.5 mL/min) Chiralcel OJ-H, t_R = 19.5 min (minor), t_R = 35.8 min (major), 91% ee.

(*R*)-2-[1-(4-Bromophenyl)-2-nitroethyl]-3-hydroxy-1,4-naphthoquinone (3f)

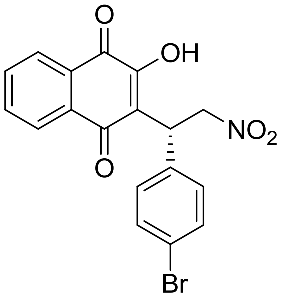

$[\alpha]^{17}_{D}$ = –17.3 (c = 1.00, CH_3COCH_3); mp 199–203 °C; ^{1}H NMR (200 MHz, $CDCl_3$) δ 8.03 (d, *J* = 7.8 Hz, 1H), 7.89 (d, *J* = 7.8 Hz, 1H), 7.72 (t, *J* = 7.8 Hz, 1H), 7.58–7.54 (m, 3H), 7.49 (d, *J* = 8.7 Hz, 1H), 7.16 (d, *J* = 8.7 Hz, 1H), 5.61–5.49 (m, 1H), 5.31–5.10 (m, 2H); ^{13}C NMR (50 MHz, $CDCl_3$): δ 37.9, 76.4, 120.3, 125.8, 126.1, 129.9, 130.2, 131.5, 131.8, 133.5, 134.8, 137.9, 156.8, 180.9, 183.8; HPLC (*n*-Hexane/iPrOH = 70/30, 254 nm, 1.5 mL/min) Chiralcel OJ-H, t_R = 14.3 min (minor), t_R = 27.1 min (major), 95% ee.

(*R*)-2-[1-(2-Fluorophenyl)-2-nitroethyl]-3-hydroxy-1,4-naphthoquinone (3g)

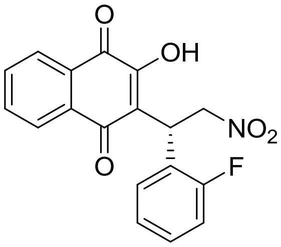

$[\alpha]^{17}_{D}$ = –21.3 (c = 1.00, CH_3COCH_3); mp 165–167 °C; ^{1}H NMR (200 MHz, $CDCl_3$) δ 8.07–8.05 (m, 2H), 7.82–7.58 (m, 2H), 7.55–7.40 (m, 2H), 7.24–6.83 (m, 2H), 5.60 (dd, *J* = 6.8 Hz, 11.0 Hz, 1H), 5.42 (m, 1H), 5.09 (dd, *J* = 7.8 Hz, 11.0 Hz, 1H); ^{13}C NMR (50 MHz, $CDCl_3$): δ 38.3, 77.2, 115.9, 119.5, 121.5, 126.7, 127.2, 128.8, 129.7, 130.2, 132.8, 133.2, 133.4, 135.9, 153.1, 161.1 (d, *J* = 245.3 Hz), 181.0, 183.7; HPLC (*n*-Hexane/iPrOH = 70/30, 254 nm, 1.5 mL/min) Chiralcel OJ-H, t_R = 10.8 min (minor), t_R = 31.5 min (major), 97% ee.

(*R*)-2-[1-(2-Bromophenyl)-2-nitroethyl]-3-hydroxy-1,4-naphthoquinone (3h)

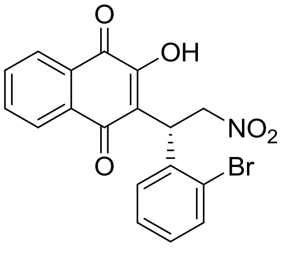

$[\alpha]^{17}_{D}$ = –39.3 (c = 1.00, CH_3COCH_3); mp 155–159 °C; ^{1}H NMR (200 MHz, $CDCl_3$) δ 8.07–8.05 (m, 2H), 7.82–7.58 (m, 2H), 7.55–7.40 (m, 2H), 7.24–6.83 (m, 2H), 5.60 (dd, *J* = 6.8 Hz,

11.0 Hz, 1H), 5.42 (m, 1H), 5.09 (dd, *J* = 7.8 Hz, 11.0 Hz, 1H); ^{13}C NMR (50 MHz, $CDCl_3$): δ 39.6, 74.6, 119.3, 126.3, 127.1, 127.8, 128.8, 129.4, 129.5, 131.2, 131.8, 132.5, 133.2, 133.4, 135.4, 136.2, 154.1, 180.6, 183.8; HPLC (*n*-Hexane/iPrOH = 70/30, 254 nm, 1.5 mL/min) Chiralcel OJ-H, t_R = 26.8 min (minor), t_R = 28.1 min (major), 95% ee.

(*R*)-2-Hydroxy-3-[2-nitro-1-(2-thienyl)ethyl]-1,4-naphthoquinone (3i)

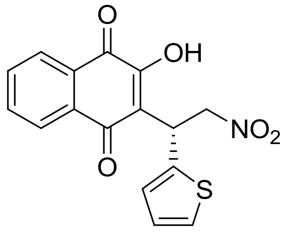

$[\alpha]^{17}_D$ = –19.7 (c = 1.00, CH_3COCH_3); mp 122–125 °C; ^{1}H NMR (200 MHz, $CDCl_3$) δ 8.04 (d, *J* = 6.8 Hz, 1H), 7.93 (d, *J* = 5.8 Hz, 1H), 7.69 (t, *J* = 6.8 Hz, 1H), 7.54 (t, *J* = 7.8 Hz, 1H), 7.05–7.03 (m, 1H), 6.95 (d, *J* = 3.9 Hz, 1H), 6.72–6.68 (m, 1H), 5.61 (dd, *J* = 5.8 Hz, 13.4 Hz, 1H), 5.46–5.35 (m, 1H), 5.21 (dd, *J* = 6.2 Hz, 13.4 Hz, 1H); ^{13}C NMR (50 MHz, $CDCl_3$): δ 34.8, 76.6, 119.9, 125.4, 126.4, 126.6, 126.9, 127.2, 129.0, 132.5, 133.4, 135.5, 138.9, 153.1, 181.0, 183.3; HPLC (*n*-Hexane/iPrOH = 85/15, 254 nm, 1.5 mL/min) Chiralcel OJ-H, t_R = 66.2 min (minor), t_R = 143.3 min (major), 95% ee.

(*R*)-2-[1-(1-Naphthyl)-2-nitroethyl]-3-hydroxy-1,4-naphthoquinone (3j)

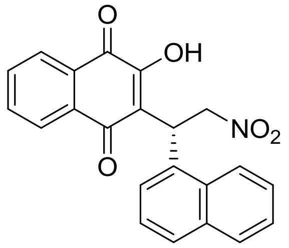

$[\alpha]^{17}_D$ = –12.2 (c = 1.00, CH_3COCH_3); mp 127–129 °C; ^{1}H NMR (200 MHz, $CDCl_3$) δ 8.48 (d, *J* = 8.5 Hz, 1H), 8.12–7.84 (m, 3H), 7.81–7.43 (m, 7H), 6.18–6.10 (m, 1H), 5.62–5.59 (m, 2H); ^{13}C NMR (50 MHz, $CDCl_3$): δ 35.5, 76.3, 120.5, 122.7, 125.2, 125.9, 126.0, 126.3, 127.1, 127.4, 128.6, 129.0, 129.2, 131.2, 132.7, 132.9, 133.3, 134.0, 135.4, 154.0, 181.0, 183.9; HPLC (*n*-Hexane/iPrOH = 85/15, 254 nm, 1.5 mL/min) Chiralcel OJ-H, t_R = 66.4 min (minor), t_R = 108.9 min (major), 99% ee.

(*R*)-2-Hydroxy-3(4-methyl-1-nitropenten-2-yl)naphthalene-1,4-dione (3k)

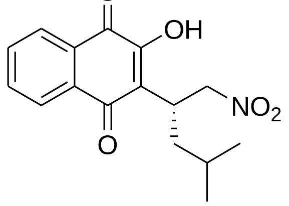

$[\alpha]^{23}_{D}$ = 39.6 (c = 1.00, CH_3COCH_3); ^{1}H NMR (200 MHz, $CDCl_3$) δ 7.92–7.60 (m, 4H), 4.94–4.91 (m, 1H), 4.57–4.53 (m, 1H), 4.09–4.02 (m, 1H), 1.89–1.77 (m, 1H), 1.46–1.35 (m, 2H), 0.91 (d, *J* = 6.2 Hz, 3H), 0.87 (d, *J* = 6.7 Hz, 1H); ^{13}C NMR (50 MHz, $CDCl_3$): δ 21.8, 23.1, 26.1, 32.8, 39.4, 77.4, 120.8, 126.3, 127.2, 129.1, 132.7, 133.2, 135.4, 154.1, 180.6, 183.8; HPLC (*n*-Hexane/iPrOH = 90/10, 220 nm, 1.5 mL/min) Chiralpak AD-H, t_R = 8.6 min (major), t_R = 11.5 min (minor), 97% ee.
